# Covalent Pectin/Arabinoxylan Hydrogels: Rheological and Microstructural Characterization

**DOI:** 10.3390/polym16202939

**Published:** 2024-10-20

**Authors:** Claudia Lara-Espinoza, Agustín Rascón-Chu, Valérie Micard, Carole Antoine-Assor, Elizabeth Carvajal-Millan, Rosalba Troncoso-Rojas, Federico Ohlmaier-Delgadillo, Francisco Brown-Bojorquez

**Affiliations:** 1 Research Center for Food and Development, CIAD, A.C., Carretera Gustavo Enrique Astiazaran Rosas No. 46, Col. La Victoria, Hermosillo 83304, Sonora, Mexico; claudialaraes@gmail.com (C.L.-E.); ecarvajal@ciad.mx (E.C.-M.);; 2 IATE, INRAE, Institut SupAgro, University Montpellier, 34000 Montpellier, France; 3 Departamento de Investigación en Polímeros y Materiales, University of Sonora, Rosales y Blvd. Luis D. Colosio, Hermosillo 83000, Sonora, Mexico; francisco.brown@unison.mx

**Keywords:** mixed hydrogel, ferulic acid, oxidative coupling, microstructural characteristics, rheological properties

## Abstract

This research aimed to evaluate the gelation process of ferulated pectin (FP) and ferulated arabinoxylan (AXF) in a new mixed hydrogel and determine its microstructural characteristics. FP from sugar beet (*Beta vulgaris*) and arabinoxylan from maize (*Zea mays*) bran were gelled via oxidative coupling using laccase as a crosslinking agent. The dynamic oscillatory rheology of the mixed hydrogel revealed a maximum storage modulus of 768 Pa after 60 min. The scanning electron microscopy images showed that mixed hydrogels possess a microstructure of imperfect honeycomb. The ferulic acid content of the mixed hydrogel was 3.73 mg/g, and ferulic acid dimer 8-5′ was the most abundant. The presence of a trimer was also detected. This study reports the distribution and concentration of ferulic acid dimers, and the rheological and microstructural properties of a mixed hydrogel based on FP and AXF, which has promising features as a new covalent biopolymeric material.

## 1. Introduction

**Figure 1:**
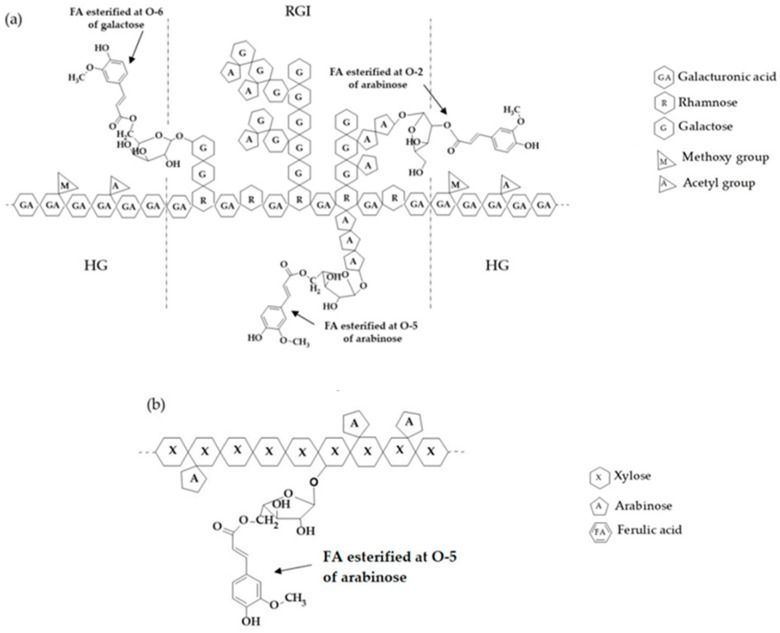
Schematic representation of the chemical structure of FP (**a**) and AXF (**b**). HG = Homogalacturonan region and RGI = Rhamnogalacturonan I region. Adapted from [11].

Ferulated pectin (FP) and arabinoxylan (AXF) are polysaccharides found in the diet as fiber from fruits and cereals, respectively [1,2]. Each biopolymer has gained significant relevance due to its wide applications and health benefits. Both polysaccharides share the structural characteristic of having covalently linked ferulic acid (FA) in their carbohydrate chains [3]. In this context, FP is a set of polysaccharides present in the cell wall of terrestrial plants and consists of linear polymers formed mainly by galacturonic acid units linked glycosidically by α-(1,4) bonds (Figure 1a). Commercially, pectin is obtained at a large scale from citrus peels and apple bagasse, although they can also be obtained from minor sources such as sweet potato (*Ipomoea batatas*), sunflower (*Helianthus annuus* L.) residues, mango peel (*Mangifera indica* L.), and sugar beet (*Beta vulgaris*) [1]. Sugar beet has been widely studied as an alternative source of pectin [4,5].

FP present in *B. vulgaris* have FA bound to galactose (O-6, position) and arabinose (O-2 position) residues in the Rhamnogalacturonan I chains (RG-I), which enables a third mechanism of gelation for pectin: oxidative coupling, in addition to the two main gelling mechanisms of pectin, which depend mainly on its degree of esterification. Thus, high-methoxy pectin requires the presence of co-solutes such as sucrose to form a gel and a pH lower than the pKa of their monomeric unit, galacturonic acid. In contrast, low-methoxy pectin require the presence of divalent ions such as calcium and a pH higher than the galacturonic acid pKa [6]. The ability of these polysaccharides to form hydrogels has become relevant in the design of matrices for the controlled release of molecules of therapeutic interest [7,8]. However, it has been observed that FP hydrogels have little gel strength. Theoretically, the latter observation is attributed to the high content of acetyl groups, apparently lower molecular mass, and long side chains [9,10].

On the contrary, ferulated arabinoxylan (AXF) are obtained from cereals, such as corn, wheat, barley, and oats. A linear chain of β forms AXF-(1,4)-D-xylanopyranose units, to which substituents of α-L-arabinofuranoses in position O-2 and O-3 are attached (Figure 1b). These polysaccharides have FA in their structure bound in the O-5 position of arabinose through an ester bond [3], allowing them to form covalent hydrogels resistant to changes in pH, temperature, and ionic strength. This ability of AXF to form hydrogels has been widely studied [2]. However, these hydrogels can have relatively extended gelling times.

**Figure 2:**
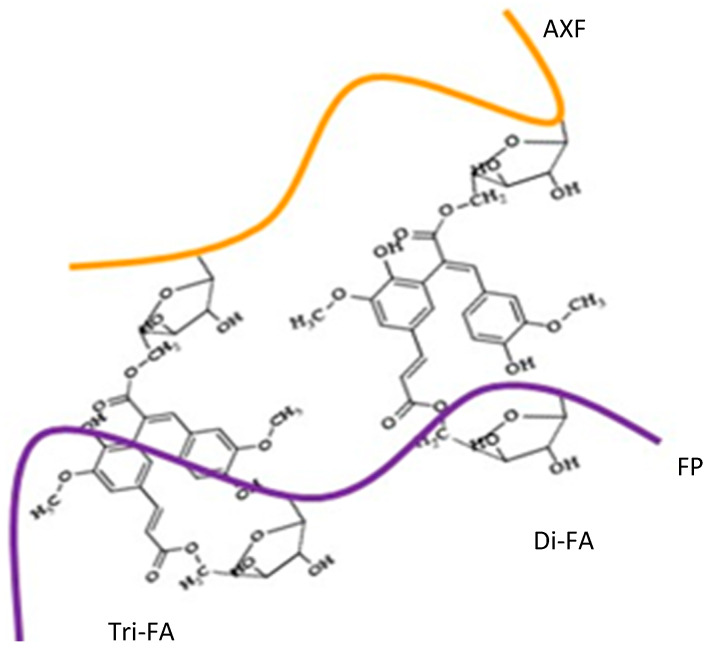
Schematics of theoretical covalent crosslinkings between the ferulated polysaccharides. A theoretical dimer of FA (di-FA) and a theoretical trimer named (tri-FA) are represented between two lines representing ferulated pectin (FP) and another representing ferulated arabinoxylan (AXF) as the expected covalent crosslinking structure in FP/AXF mixed hydrogel. Adapted and modified from [11].

Due to the feature that both polysaccharides have covalently linked FA in their structures, the ability to form FA dimers (di-FA), where two units of ferulic acid residues are covalently linked, and trimers (tri-FA), where three units of ferulic acid residues are covalently linked, are schematically represented in Figure 2 as theoretical structures between both chains, and their formation is determined in the present work. A mixed hydrogel of such nature has never been reported yet. It is unknown whether the structural differences prevent the coupling of FA from FP and FA from AXF. In this sense, this work proposes a new composite material. The characterization will eventually envision potential applications for the food industry and biomedicine. Thus, the present investigation aims to determine the rheological and microstructural characteristics of FP/AXF mixed hydrogels.

## 2. Materials and Methods

### 2.1. Plant Material Preparation and Reagents

Sugar beet roots (*B. vulgaris*) were grown and supplied by the Agronomy Faculty of Sonora University (coordinates: 29_00048″ NL, 111_08007″ WL, and 151 masl) for the obtention of FP. Briefly, samples were washed with tap water and soap to remove dirt and then were sliced in pieces 1 cm thick in a slicing machine (Hobart, Troy, OH, USA) and dried in an oven (Enviro-Pak Inc., Clackamas, OR, USA) at 50 °C with a forced airflow of 2.5 m^3^ s^−1^ for 20 h. Finally, the samples were kept at −40 °C until use.

Maize (*Z. mays*) bran was provided by an agricultural company in Sinaloa, Mexico, to extract AXF. The maize bran was separated from the kernel by a mechanical process. Neither chemical nor enzymatic treatments were used.

Commercial laccase from *Trametes versicolor* (0.99 U/mg) was obtained from Sigma-Aldrich Chemical Co. (St. Louis, MO, USA). All the reagents used were of analytical grade.

### 2.2. FP and AXF Extraction

FP was extracted following the methodology reported by Li et al. [12], with minor variations. Briefly, 150 g of dried sugar beet was suspended in 1.5 L of 0.1 M hydrochloric acid (1:10 *w*/*v*), and the pH was adjusted to 1.5. The mixture was homogenized, heated on a plate with stirring at 85 °C for 1 h, and allowed to cool at room temperature. Next, the mixture was filtered through a nylon cloth (pore size 60 μm) and centrifuged at 10,000× *g* for 20 min in a centrifuge (Thermo Scientific™, Waltham, MA, USA). The samples were ethanol-precipitated (2:1 *v*/*v*) and dried by solvent exchange with 80% *v*/*v* ethanol, absolute ethanol, and acetone. Once dry, the samples were ground in a coffee grinder (Hamilton Beach^®^, Glen Allen, VA, USA) to a fine powder and stored in a sealed container, in the dark at room temperature until use.

AXF was extracted as characterized by Morales-Burgos et al. [13]. Briefly, maize bran was milled, suspended in ethanol, and agitated on a rotatory shaker for 12 h at 25 °C. The ethanol-treated bran was filtered and subjected to enzyme inactivation (boiling water for 30 min). Afterward, maize bran was recovered by filtration and then treated with a NaOH solution at 25 °C in darkness under shake conditions. Then, the mixture was filtrated to eliminate residual solids. The filtrate was centrifuged, and the obtained supernatant was acidified to pH 4 with HCl and centrifuged. Finally, the supernatant was precipitated in ethanol for 4 h at 4 °C, then recovered and dried by solvent exchange. It was stored as powder in a sealed container in the dark at room temperature until use.

### 2.3. FP/AXF Mixed Hydrogel Formation

Mixed hydrogels were formed, keeping FA from each polysaccharide in a 1:1 proportion to a final total concentration of 26 mg/mL or 2.6% (*w*/*v*). Polysaccharide solutions were dissolved in acetate buffer pH 5.5, separated, and then mixed, and laccase enzyme was added as a crosslinking agent (83.33 nkat enzyme/mg of FA). FP and AXF solutions at 3% and 2.3% (*w*/*v*) were prepared as mentioned above and used as controls. The hydrogels were left to form at 25 °C for 2 h in the dark.

### 2.4. Phenolic Acid Content

The FA, di-FA, and tri-FA content were analyzed by high-performance liquid chromatography. The mixed hydrogels (50 mg) were saponified (2 mL of 2 N NaOH, 100 rpm, 35 °C, and 2 h in darkness). The pH was adjusted to 2.0 using 4 N HCl. Phenolic acid was recovered twice in 5 mL of diethyl ether and evaporated under N_2_ flux at 40 °C. The extract was resuspended in 1 mL methanol/water (50:50 *v*/*v*) and filtered (0.45 µm, Millipore, St. Louis, MO, USA). The samples were injected in an HPLC (Waters™, Milford, MA, USA) equipped with a C18 column (Alltech Inc., Lexington, KY, USA; 250 mm × 2.6 mm), previously filtered in a 0.45 µm membrane [13,14]. Detection was performed at 320 nm and 3,4,5-trimethoxycinnamic acid (TMCA) was used as internal standard.

### 2.5. Rheological Measurements

The rheological properties of the mixed hydrogels were determined by a small-amplitude oscillatory shear using a strain-controlled rheometer (Discovery HR-2 rheometer, TA Instruments, New Castle, DE, USA), with a parallel-plate geometry (40 mm in diameter), following the methodology proposed by Vansteenkiste et al. [14]. The gelation process of the polysaccharide mixture was monitored for 1 h at 25 °C, following the storage (G′) and loss (G”) modulus. During gelation rheological measurements, a silicone oil layer was used to cover the polysaccharide mixture to prevent evaporation. All measurements were performed at a frequency of 0.25 Hz and 5% strain (linearity range of viscoelastic behavior). After the gelation process, a mechanical sweep was conducted in the 0.01–10 Hz range, with 5% strain and a temperature of 25 °C.

### 2.6. Microstructural Characterization

The mixed hydrogels were frozen to −20 °C and then lyophilized at −80 °C with 0.008 mbar in a Freezone 6 freeze drier (Labconco, Kansas, MO, USA). The obtained hydrogels were then coated with a thin layer of gold. The microstructure of the mixed hydrogels was observed via scanning electron microscopy (SEM) using a microscope (JEOL 5410LV, JEOL, Peabody, MA, USA) with an accelerating voltage of 20 kV.

### 2.7. Statistical Analysis

All results are expressed as mean values ± standard deviation based on triplicate measurements. A one-way analysis of variance (ANOVA) was performed to determine significant differences between the respective values, and averages were compared by the Tuckey–Kramer test with a *p* ≤ 0.05. The statistical software NCSS 12 (Setup_v12_0_18.exe, 30 August 2021) was used.

## 3. Results and Discussion

### 3.1. Chemical Characterization of FP and AXF

The chemical composition of sugar beet FP is presented in Table 1. FP showed a galacturonic acid content of 55 g/100 g of pectin. This compound was the major component present in the structure of FP. The total neutral sugars accounted for 21.6 g/100 g of pectin, mainly rhamnose (6.3 g/100 g), galactose (7.1 g/100 g), and arabinose (3.3 g/100 g), which are the main sugars reported for the RG-I region in the structure of FP [9,10,15]. In addition, other neutral sugars such as xylose, glucose, fucose, and mannose were detected in lower amounts.

The FA content was 5.5 mg/g of pectin, and the presence of di-FA was 0.26 mg/g. The presence of tri-FA was not detected. The studied FP also showed a high content of protein (10.3 g/100 g of pectin), which was higher than those generally reported in FP obtained from sugar beet [16]. The latter, due to proteases, were not used in this extraction process. The ash content was 2.13 g/100 g of pectin. Pectin also showed a degree of methylation and acetylation at 57.4% and 26.1%, respectively (Table 1).

The AXF used in this investigation was previously characterized by Morales-Burgos et al. [13] and presented an arabinose-to-xylose ratio of 0.72 and an FA and di-FA content of 7.18 and 0.44 mg/g AXF, respectively.

### 3.2. Ferulic Acid, Dimer, and Trimer Content in Mixed Hydrogels

To cast hydrogels where FA was in equal amounts from each polysaccharide, a mix of FP at 1.5% and arabinoxylan at 1.15% were mixed to a final total polysaccharide percentage of 2.6%. As FA content is the common residue for all hydrogels, polymers’ concentration and laccase were fixed as a function of FA (83.33 nkat enzyme/ mg of FA). To some extent, the latter seemed to be the logical way to control laccase, as FA was the main substrate present. In Table 2, the resulting phenolic acid content in the mixed hydrogels are shown. Initially, the FP/AXF mixture (before gelation) exhibited an FA concentration of 6.2 ± 0.3 mg/g, while the mixed hydrogel (after gelation) showed a total FA content of 3.7 ± 0.2 mg/g. The FA content diminished consistently with the crosslinking formation process where di-FA and tri-FA were generated. It can be noted how the proportions of total FA dimer and trimer content increased after the gelling process and correlated with the oxidation of FA by the action of the enzyme laccase. Only 40% of the initial FA was oxidized (2.48 mg/g), and 60% remained free FA, corresponding to the 3.7 mg FA/g dw obtained in the mixed hydrogel.

Similarly, Mendez-Encinas et al. [17] studied AXF hydrogels and reported that laccase action oxidized only 35% of FA original content. This percentage was slightly lower than that found in this investigation for the mixed hydrogels. On the contrary, Martinez-Lopez et al. [18] reported that 70% of the initial FA was oxidized to form dimers and trimers of FA in AXF microspheres. These discrepancies may be due to further structural differences such as molecular weight, neutral-sugar ratio, viscosity, enzyme concentration, and laccase properties.

**Figure 3:**
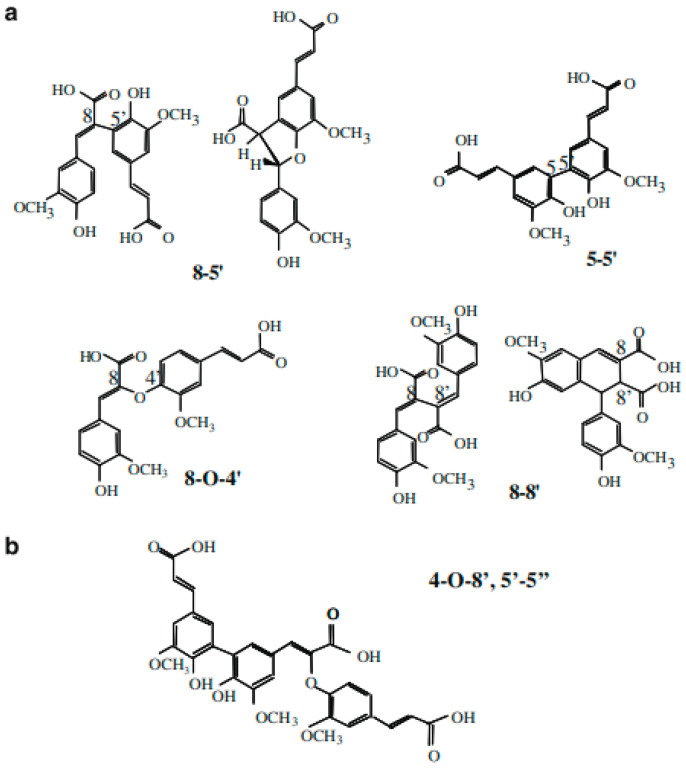
Schematic chemical structures of dehydrodimers of ferulic acid (di-FA) (**a**) and a dehydrotrimer of ferulic acid (tri-FA) (**b**). Adapted from [23].

**Figure 4:**
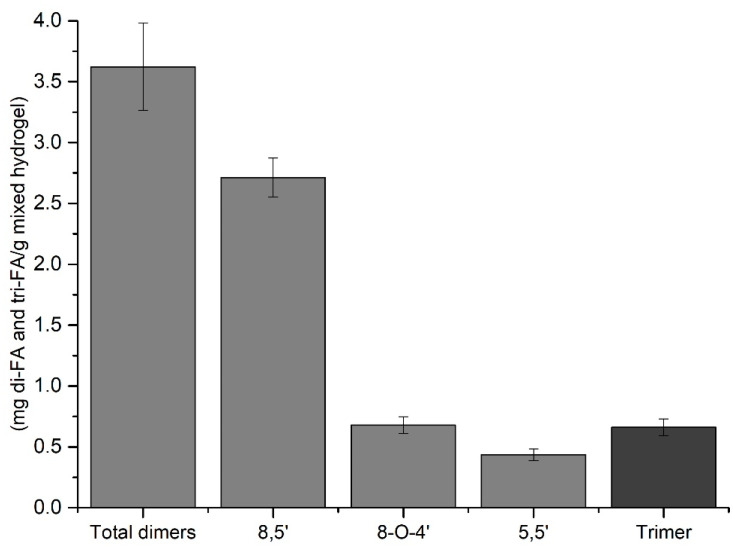
Dimers (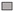) and trimer (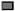) of ferulic acid after gelation in the mixed hydrogel of FP and AXF. Results are presented as mean ± SD based on triplicate measurements.

Nevertheless, remaining free FA can represent an advantage in mixed hydrogels since it has been widely reported that FA has antioxidant capacities. Figure 3 shows a schematic representation of the structure of the most-reported dimers and a trimer. Their names derive from the carbon position of the ferulic acid units that participate in the covalent linkage. In Figure 4, the results for the distribution and concentration of the di-FA and a tri-FA in the mixed hydrogels after FA oxidation are shown. From the total number of the newly formed dimers, the 8-5′ structure was the predominant form after gelation, representing 69% of the total content of dimers, while the 8-O-4′ and 5-5′ structures represented 19% and 12%, respectively. Several authors report the 8-5′ dimer as the main isomer in FP [19,20] and AXF hydrogels [13]. Moreover, a similar pattern was recently observed in the data obtained by Ohlmaier-Delgadillo et al. [11] for a mixed hydrogel. This hydrogel was composed of a low-methoxy FP obtained from a sugar beet solid waste and AXF from maize dried distiller’s grains, where the dimers 8-5′, 8-O-4′, and 5-5′ were found, with the 8-5′ isomer being the main one. The former di-FA values confirmed the results presented in this work. In this regard, several studies have evaluated the antioxidant activity of AXF [21,22] and AXF hydrogels [17]. Mendez-Encinas et al. [17] studied the antioxidant capacity of AXF hydrogels and observed that FA exhibited antioxidant capacity even after gelling. These authors reported a concentration of 3.52 mg/g in AXF hydrogel.

Furthermore, the antioxidant activity of the FA oligomers formed after the dimerization of FA has also been reported by several authors [24,25,26]. These authors infer that the antioxidant activity is superior in FA dimers than in its substrate (FA). In this context, Garcia-Conesa et al. [24] studied the antioxidant capacity of ferulic acid dimers, chemically synthesized, concluding that their antioxidant capacity is dependent on the free phenolic hydroxyl groups present in their structure as well as the nature of the dimer formed. These same authors also studied the antioxidant activity of ferulic acid dimers isolated from wheat bran, inferring that ferulic acid dimers, specifically 8-O-4′, showed better antioxidant activity than free ferulic acid [25]. The FA dimers reported in this study for a mixed hydrogel could exert additional antioxidant activity on the abovementioned FA content. However, further research is required to support this hypothesis conclusively.

The major presence of the 8-5′ dimer is a desirable feature for our high-methoxy FP, given its spatial conformation contributes to a more accessible way for the crosslinking between polysaccharide chains and the elasticity of the hydrogel. In addition, this isoform type is reported to be thermodynamically more stable [2]. Furthermore, a tri-FA was also detected in the mixed hydrogel (Figure 1). This isomer was also detected and identified for AXF from maize (*Z. mays*) and sugar beet (*B. vulgaris*) pectin. Indeed, 8-O-4′, 5-5′ was the first tri-FA reported in natural maize bran [27,28] and AXF hydrogels [29]. In addition, more regioisomers of tri-FA have been identified as naturally occurring in maize and sugar beet [30,31]. The latter is an opportunity for new research in mixed hydrogels and for new FA trimers, tetramers, and pentamers proposed in cereal cell walls, may also be present in this new proposed material for the spatial distribution of pectin RGI; and RGII “hairy” regions may affect the FA residues’ encounter with the final gel scaffolding.

### 3.3. Rheological Characterization of Mixed Hydrogels

**Figure 5:**
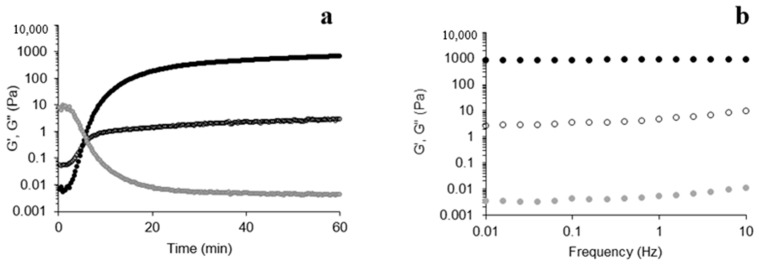
Rheological kinetics of 1.5 and 1.15% (*w*/*v*) FP and AXF mix dispersion during gelation at 0.25 Hz and 5% strain (**a**) and mechanical spectra of mixed gel after 1 h gelation at 5% strain (**b**). (*G’*●, *G*”○, tan *δ*●). Experiments were performed at 25 °C.

The G’ (storage modulus) and G” (loss modulus) of the mixed solution (FP 1.5%; AXF 1.15%) (*w*/*v*) were monitored via oxidative gelation by laccase over time. The rheological results are shown in Figure 5a. At the beginning of the gelling process, G” was higher than G’, but an increment in G’ occurred rapidly and prevailed over G” until the end. The gelation time of the mixed hydrogel was ~4 min, which was extrapolated from the crossover of G’ and G” (tan δ = 1), indicating the sol/gel transition point. The mixed hydrogel showed maximum G’ and G’‘ values of 768 and 3.30 Pa, respectively, with a final tan δ value (G”/G’) of 0.0043 at 60 min. The increment in G’ over time corresponds to di-FA and tri-FA formation; evidently, the modulus of elasticity or storage is directly proportional to the number of crosslinks, which in turn influences the strength of the hydrogel. Also, as mentioned above, the predominant content of 8-5′ di-FA contributed to the elasticity observed in the hydrogel.

The mechanical spectrum of the mixed hydrogel is shown in Figure 5b, showing a typical behavior of a solid-like material with a prevalence of G’ over G” and a lineal G’ independent of frequency and a much smaller G” dependent on frequency [32]. Also, the hydrogels showed stable behavior under the tested conditions.

The general results of small oscillatory shear rheology and density of crosslinks values [33], including AXF and FP hydrogels, are summarized in Table 3. The G’ value was statistically higher for the arabinoxylan hydrogel control, and it can be observed that the mixed hydrogels presented a higher storage modulus concerning the pectin hydrogel. Regarding G”, its value was lower for arabinoxylan hydrogel, indicating less viscous behavior at the end of the 60 min run. Interestingly, the mixed-hydrogel G’ and density of crosslink values are midway, resembling more of a proportionate behavior although not linear. The rheology parameters reflect the 1:1 FA from each polymer and the multiple interactions of their chains. This may explain why the G’ and the density of crosslinks values of the mixed gels are close to the middle value corresponding to AXF and FP hydrogels per the logarithmic additivity rule.

Complementarily, the tan δ data showed no significant difference between arabinoxylan and the mixed hydrogels (0.0021 and 0.0043, respectively). Conversely, the FP hydrogel showed tenfold a value for tan δ (0.012), which was related to a higher G” observed for this hydrogel. The latter suggests that some FP chains were only dispersed in the polymeric network and did not participate in the crosslinking scaffold, contributing mainly to the viscous module. In sum, the values of tan δ for the AXF hydrogels and mixed FP/AXF hydrogels denote higher participation of the polysaccharide chains in the polymer network than the FP hydrogels. The “hairy” regions play a significant role in this rheological observation and the resulting repulsion due to a pH value of 5.5 favoring charged residues.

Structural properties such as the xylose/arabinose ratio in AXF [2], the number of lateral chains in pectin [34], and physicochemical properties such as FA content, molecular weight, and the viscosity of polysaccharides have a notable effect on the gelling capacity of hydrogel matrices [2,11].

In this context, in a study conducted by Schooneveld-Bergmans et al. [35], the oxidative crosslinking of AXF from both wheat bran and flour was evaluated, showing that both polysaccharides participated in the crosslinking and had a synergistic effect on viscosity; nevertheless, in our study, the results observed do not present this behavior. So, an apparent interaction of both the polysaccharides (FP and AXF) on their rheology can be noted as logarithmic additivity. Also, FA has an important effect on a polysaccharide’s physical and functional characteristics.

### 3.4. Microstructure of Hydrogels

**Figure 6:**
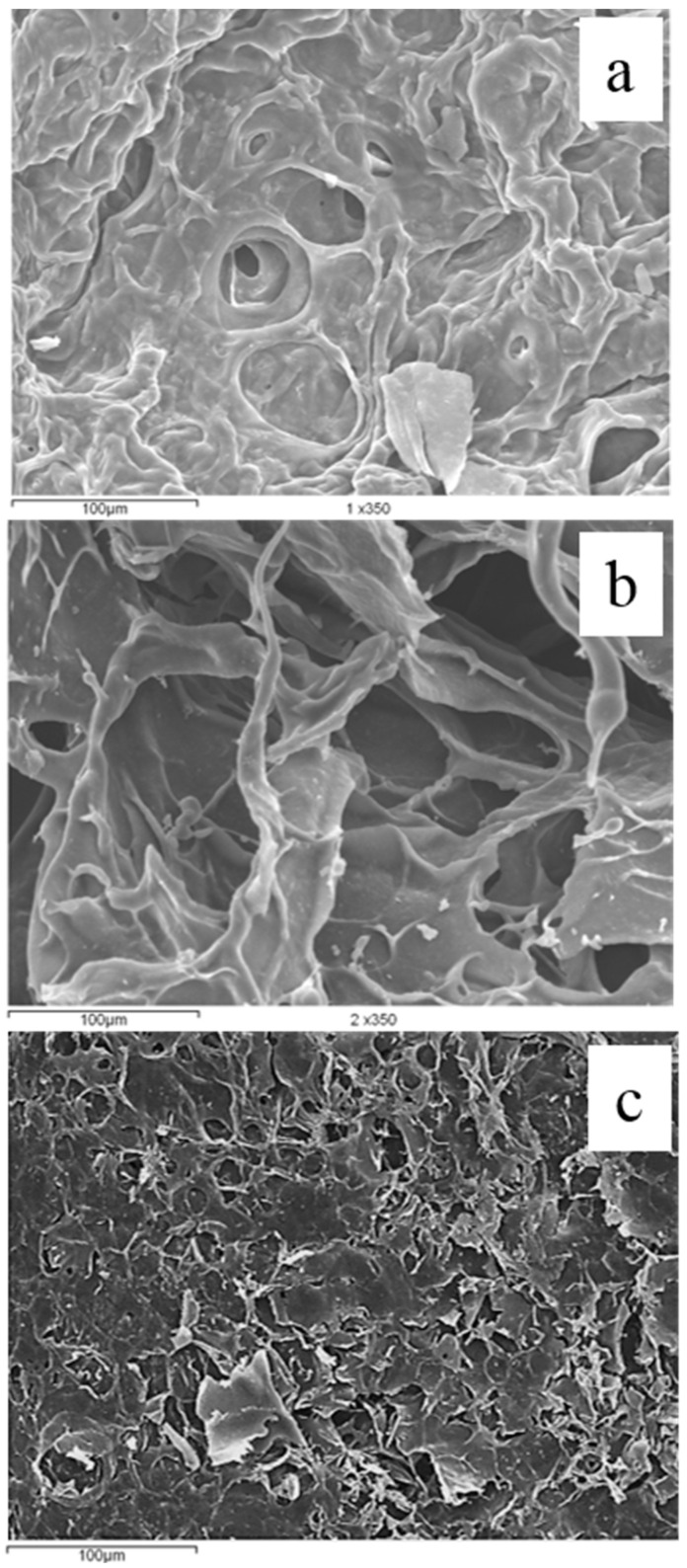
SEM images of Arabinoxylan gel (2.3%) (**a**), pectin gel (3%) (**b**), and mixed gel (pectin 1.5%/AX 1.15%) (**c**). Magnification 350×.

The microstructure of the hydrogels, evaluated at a scale of 100 µm by SEM, is shown in Figure 6. The AX hydrogel presented a regular, highly porous microstructure resembling an imperfect honeycomb. This microstructure has been observed widely in other investigations in arabinoxylan from maize [14]. In the FP hydrogel, a highly porous microstructure was also observed, but its cavities (alveolate) were considerably irregular and resembled an imperfect honeycomb. The observed microstructure agrees with the reported elsewhere [36,37].

The SEM image of the mixed hydrogel conserved a porous microstructure with somewhat comparable cavities. Compared to pure pectin or arabinoxylan hydrogels, a more compact microstructure can be observed. Similarly, previous studies of low-methoxy pectin hydrogels showed a comparable microstructure [37]. The evidence in our study suggests that ferulated pectin covalent crosslinking in the microstructure observed in the hydrogels resembles an imperfect honeycomb more instead. Further research is needed to conclude this fully. The current data suggest that different polysaccharide ratios and FA content might be critical to designing materials with different pore sizes and alveoli. In this regard, research has been undertaken to fully assess the potential for bioactive-compound encapsulation in combination with probiotics. Hypothetically, pore size and alveolate pattern regulation with polysaccharide ratios and FA content would be feasible. Nevertheless, we are not at that point yet.

## 4. Conclusions

The FP/AXF mixed hydrogel was successfully created via oxidative coupling, showing a higher storage modulus than FP hydrogel. Through tan delta, it could be inferred that a close interchain interaction between both polysaccharides affects rheological properties. The microstructure of the mixed hydrogels showed microstructural characteristics for both polysaccharides resembling honeycombs. A more compact structure gives this material an advantage over pectin or arabinoxylan hydrogels by separating the design of bio-composites with suitable properties for encapsulating bioactive compounds. This investigation is one of the first to report the FA dimers, trimer distribution, and the rheological and microstructural characteristics of an FP/AXF mixed hydrogel with promising features as a new covalent biopolymeric material.

## Figures and Tables

**Table 1:** Chemical characterization of FP from sugar beet.

Compound	Content
Galacturonic acid ^1^	55.0 ± 3
Rhamnose ^1^	6.3 ± 0.5
Arabinose ^1^	3.3 ± 0.4
Xylose ^1^	1.4 ± 0.2
Galactose ^1^	7.1 ± 0.5
Glucose ^1^	1.0 ± 0.01
Mannose ^1^	1.3 ± 0.2
Fucose ^1^	1.2 ± 0.1
Total neutral sugars ^1^	21.6 ± 0.9
Protein ^1^	10.3 ± 0.5
Ash ^1^	2.13 ± 0.06
DM ^1^	57.4 ± 4.1
DA ^1^	26.1 ± 2.4
Ferulic acid ^2^	5.5 ± 0.1
Diferulic acid ^2^	0.26 ± 0.03

DM, degree of methylation; DA, degree of acetylation. ^1^ Results expressed in g/100 g pectin; ^2^ Expressed in mg/g pectin.

**Table 2:** FA, FA dimer, and FA trimer content in FP/AXF mixed before and after 2 h of gelation.

	FA	Di-FA	Tri-FA	FA Oxidized ^1^
	(mg/g)			(%)
Before gelation	6.2 ± 0.3	0.34 ± 0.03	0.010 ± 0.002	-
After gelation	3.7 ± 0.2	3.6 ± 0.3	0.66 ± 0.07	40

^1^ FA oxidized, calculated from the initial total FA oxidized during the gelling process.

**Table 3:** Storage modulus (G’), loss modulus (G”), and tan delta (tan δ) of AXF, FP, and FP/AXF mixed gels.

	G’ (Pa)	G” (Pa)	Tan δ (G”/G’)	Density of Crosslinks (mol/cm^3^) × 10^−7^
AXF hydrogel (2.3%)	1540 ± 280 ^b^	2.53 ± 0.26 ^a^	0.0021 ± 0.0007 ^a^	6.21
FP hydrogel (3%)	425 ± 11 ^a^	4.64 ± 0.16 ^c^	0.012 ± 0.001 ^b^	1.71
FP/AXF (2.6%) mixed hydrogel	768 ± 2 ^a^	3.30 ± 0.04 ^b^	0.00430 ± 0.00005 ^a^	3.10
(log additivity rule) [33]	809	3.4	0.0050	3.3

Values are presented as mean ± SD of triplicate analyses. Different letters in the same column express differences (*p* < 0.05) between the hydrogels.

## Data Availability

Publicly available datasets were analyzed in this study. These data can be found here: https://ciad.repositorioinstitucional.mx/jspui/. (accessed on 5 September 2024).
